# Supplementary material for: A revised road map for the commitment of human cord blood CD34-negative hematopoietic stem cells
Source: Nat Commun. 2018 Jun 6;9:2202. doi: 10.1038/s41467-018-04441-z (PMC5989201; doi:10.1038/s41467-018-04441-z)
Supplement: Supplementary file 1 — Supplementary Information [file 41467_2018_4441_MOESM1_ESM.pdf]

Supplementary Information

A Revised Road Map for the Commitment of Human Cord Blood CD34-negative Hematopoietic Stem Cells

Sumide et al.

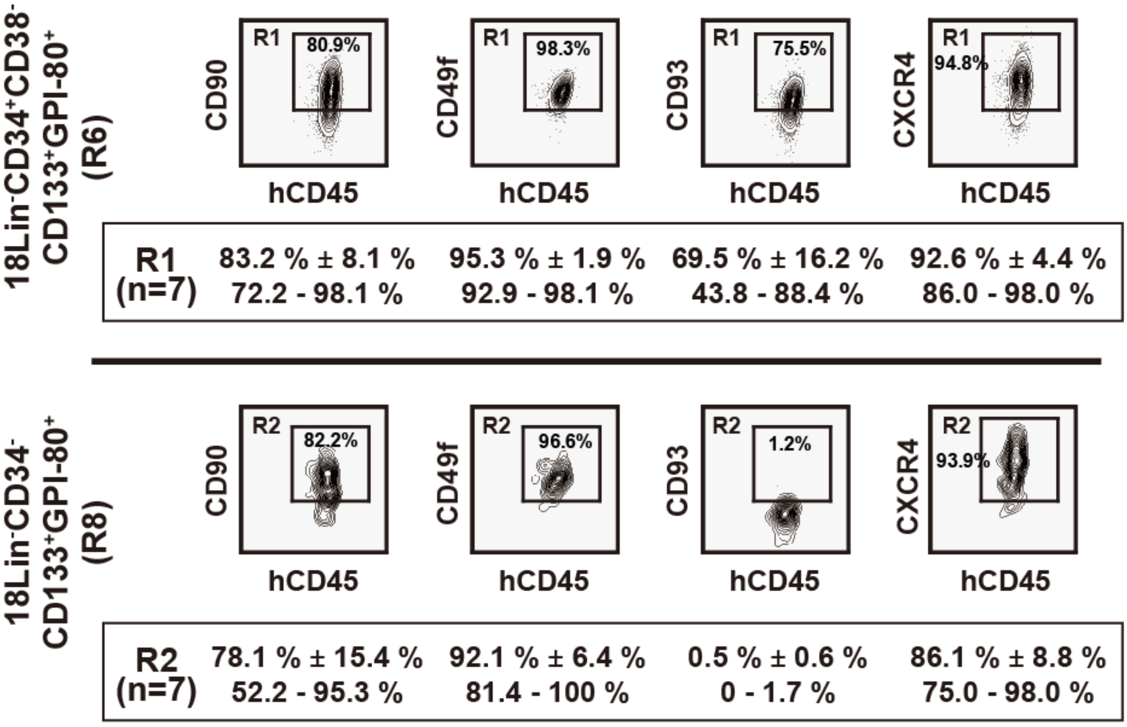

Supplementary Figure 1. Analyses of the expressions of CD90, CD49f, CD93 and CXCR4 on the 18Lin$^-$CD34$^+$CD38$^-$CD133$^+$GPI-80$^+$ and 18Lin$^-$CD34$^-$CD133$^+$GPI-80$^+$ cells.

The surface expressions of CD90, CD49f and CD93 which were recently reported as positive markers for primitive human CD34$^+$ and CD34$^-$ HSCs, and chemokine receptor CXCR4 on the 18Lin$^-$CD34$^+$CD38$^-$CD133$^+$GPI-80$^+$ (upper panel) and 18Lin$^-$CD34$^-$CD133$^+$GPI-80$^+$ cells (lower panel) were analyzed. The representative FCM data obtained from single CB unit are presented. Percentages of positive cells of each marker are depicted in the figures. We analyzed 7 single CB units, and the detailed FCM data, including the mean ± S.D. and ranges of proportions of positive cells in each gate are depicted in the figures. The numbers of cells (events) analyzed for each antigen ranged from 500 to 12,000 in 18Lin$^-$CD34$^+$CD38$^-$CD133$^+$GPI-80$^+$ cells (R6 gate in **Figure 1**) and 50 to 4,500 in 18Lin$^-$CD34$^-$CD133$^+$GPI-80$^+$ cells (R8 gate in **Figure 1**).

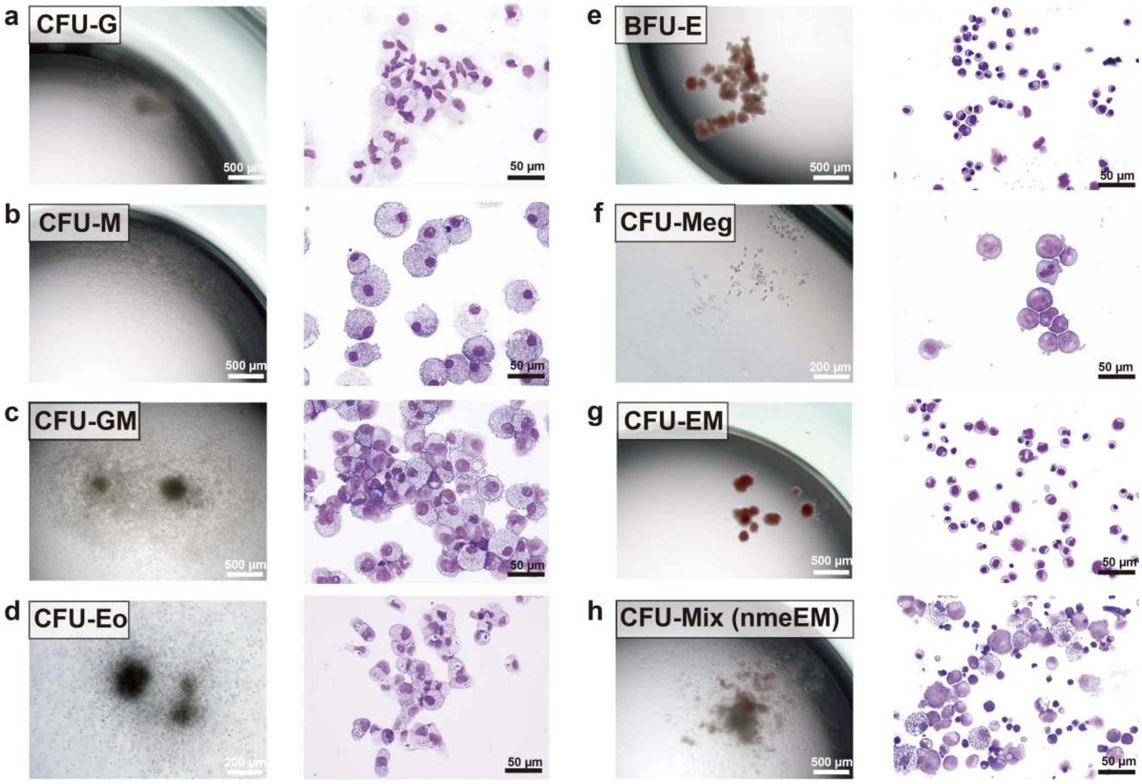

Supplementary Figure 2. Macroscopic and microscopic images of representative colonies derived from single cells.

Single 18Lin$^-$CD34$^+$CD38$^-$CD133$^+$GPI-80$^{+/-}$ or 18Lin$^-$CD34$^-$CD133$^+$GPI-80$^{+/-}$ cells were sorted into a 96-well flat-bottomed plate by an FACSAriaII using the ACDU. Each well contained 50 µL of our standard methylcellulose culture medium containing 30 % FCS and 6 cytokines (TPO, SCF, IL-3, GM-CSF, G-CSF and Epo), as previously reported[1-3]. After single-cell sorting, 50 µL of culture medium was carefully added to each of the well. Plates were cultured for 10 to 14 days and were then investigated under an inverted microscope. (**a**) CFU-G containing neutrophils, (**b**) CFU-M containing macrophages, (**c**) CFU-GM containing neutrophils, macrophages and/or eosinophils, (**d**) CFU-Eo containing eosinophils, (**e**) BFU-E containing erythroblasts/erythrocytes, (**f**) CFU-Meg containing megakaryocytes, (**g**) CFU-EM

containing erythroblasts/erythrocytes and megakaryocytes, and (**h**) CFU-Mix (nmeEM) containing neutrophils, macrophages, eosinophils, erythroblasts/erythrocytes and megakaryocytes. Macroscopic photos were taken at x40 or x100 magnification, while microscopic photos were taken at x400 magnification. Scale bars are depicted in each of the figures.

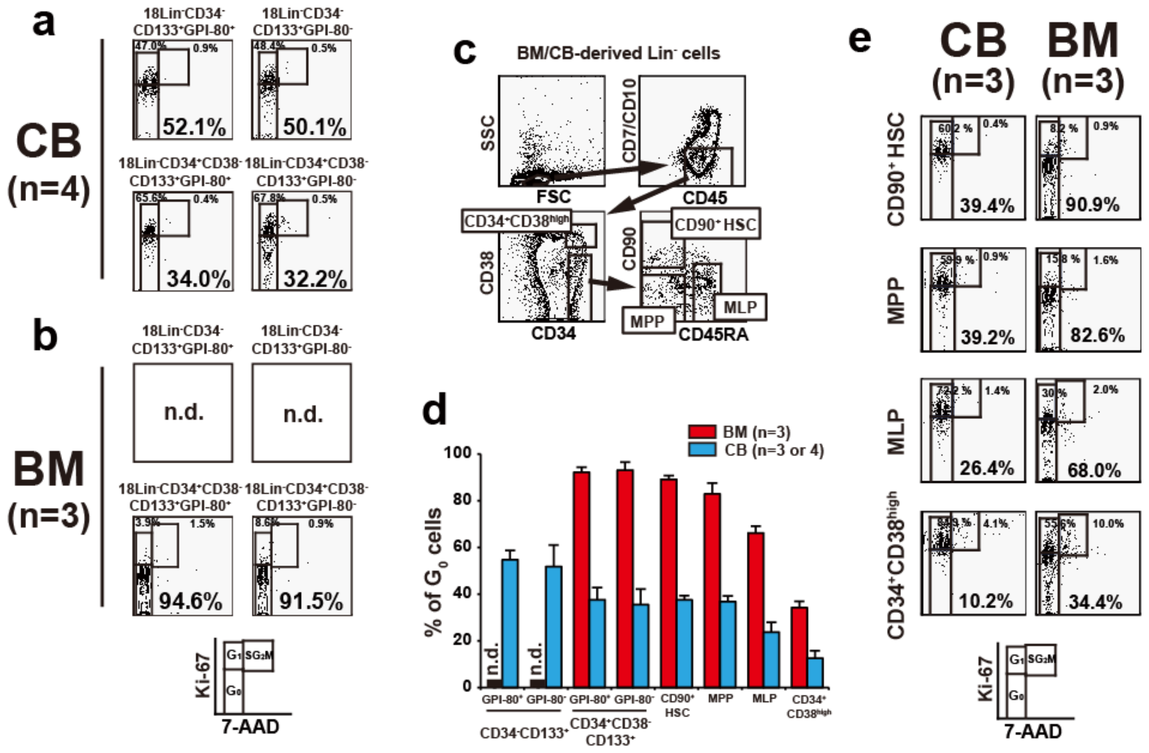

Supplementary Figure 3. A cell cycle analysis of purified CB-derived 18Lin$^-$CD34$^+$CD38$^-$CD133$^+$GPI-80$^{+/-}$ cells (CD34$^+$GPI-80$^{+/-}$ HSCs) and 18Lin$^-$CD34$^-$CD133$^+$GPI-80$^{+/-}$ cells (CD34$^-$GPI-80$^{+/-}$ HSCs) in comparison to CB-derived CD90$^+$ HSC, MPP, MLP and CD34$^+$CD38^{high} cells and BM-derived CD34$^+$GPI-80$^{+/-}$ HSCs, CD90$^+$ HSC, MPP, MLP and CD34$^+$CD38^{high} cells.

(**a**) (**b**) CB- and BM-derived lineage-negative cells were stained with various monoclonal antibodies as shown in **Figures 1a** to **f**. These cells were fixed and permeabilized using the Cytofix/Cytoperm kit (BD Biosciences). Cells were then stained with an anti-Ki-67 antibody and 7-AAD. The distribution of G_0/G_1/SG_2M-phase cells in the each cell fraction is shown. n.d., not detected. (**c**) Sorting strategy for the isolation of CD90$^+$ HSC, MPP, MLP and CD34$^+$CD38^{high} cells. (**d**) Percentages of G_0-phase cells in the depicted fractions of cells. The red bars represent BM, and the blue

bars represent CB. n.d., not detected. (**e**) The distribution of the G_0/G_1/SG_2M-phase cells in each cell fraction of CB or BM is shown.

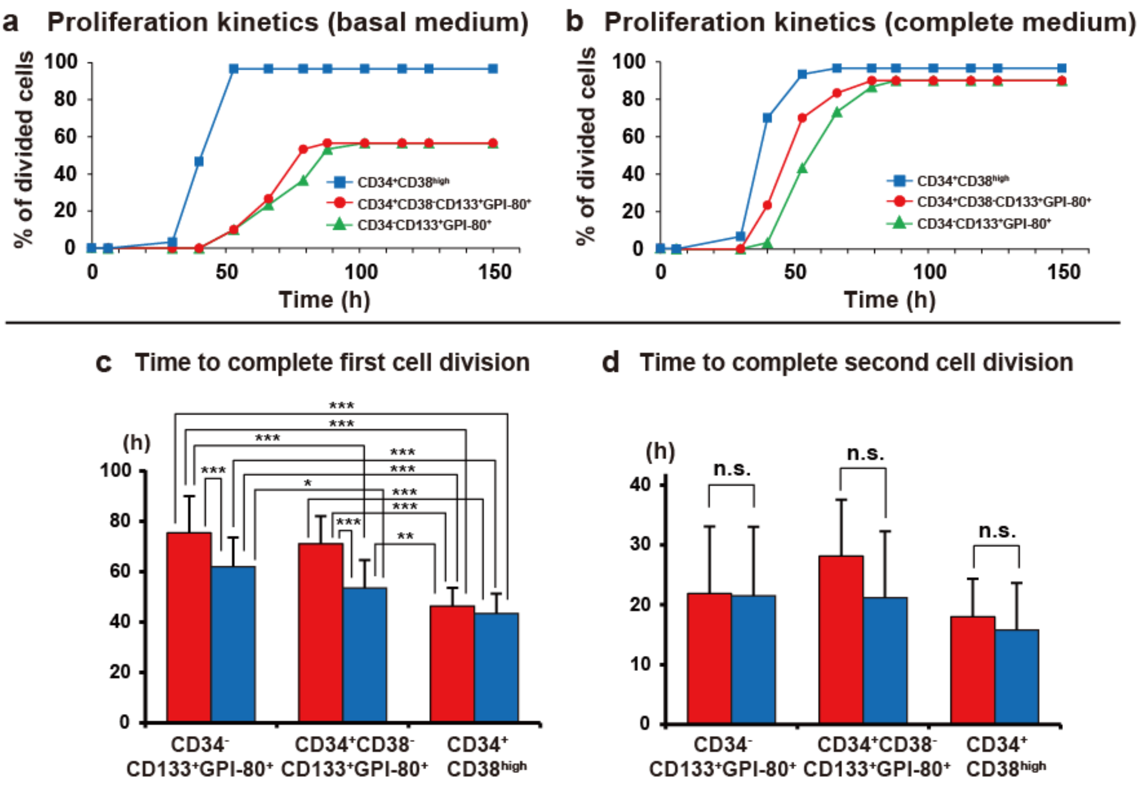

Supplementary Figure 4. A single-cell proliferation assay of 18Lin$^-$CD34$^+$CD38$^-$CD133$^+$GPI-80$^+$ cells (CD34$^+$ HSCs) and 18Lin$^-$CD34$^-$CD133$^+$GPI-80$^+$ cells (CD34$^-$ HSCs).

Single 18Lin$^-$CD34$^+$CD38$^-$CD133$^+$GPI-80$^+$ (CD34$^+$ HSC), 18Lin$^-$CD34$^-$CD133$^+$GPI-80$^+$ (CD34$^-$ HSC) and 18Lin$^-$CD34$^+$CD38^{high} (HPC, as a control) cells were cultured in (**a**) basal medium (containing TPO and SCF) or (**b**) complete medium (containing 10 % FCS and TPO, SCF, FL, G-CSF, GM-CSF, IL-3 and IL-6) for up to 144 h. The cumulative first cell division time of 18Lin$^-$CD34$^+$CD38$^-$CD133$^+$GPI-80$^+$ cells (red line), 18Lin$^-$CD34$^-$CD133$^+$GPI-80$^+$ cells (green line) and 18Lin$^-$CD34$^+$CD38^{high} cells (blue line) was analyzed. (**c**) The mean time to complete the first cell division of each class of HSCs/HPC in the basal medium (red bar) and complete medium (blue bar) (mean ± S.D., n = 30 well/group, * $p < 0.05$, **$p < 0.01$, *** $p < 0.001$, Tukey's multiple

comparison procedure). (**d**) Mean time required for completing the second cell division from the first one in each class of HSCs/HPC in the basal medium (red bar) and complete medium (blue bar) (mean ± S.D., n = 30 well/group, n.s., not significant, Mann-Whitney U test).

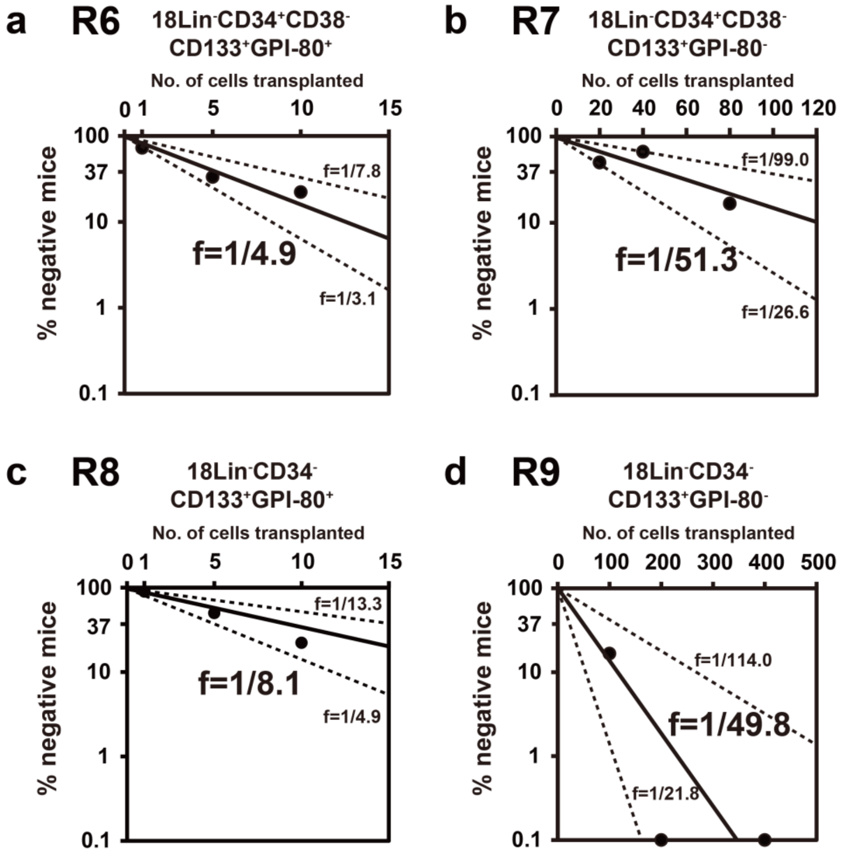

Supplementary Figure 5. A limiting dilution analysis (LDA).

(**a** - **d**) In order to estimate frequencies of SRCs in the CB-derived 18Lin$^-$CD34$^+$CD38$^-$ CD133$^+$GPI-80$^{+/-}$ and 18Lin$^-$CD34$^-$CD133$^+$GPI-80$^{+/-}$ fractions, various numbers of 18Lin$^-$CD34$^+$CD38$^-$CD133$^+$GPI-80$^{+/-}$ and 18Lin$^-$CD34$^-$CD133$^+$GPI-80$^{+/-}$ cells (precisely shown in **Supplementary Data 1**) were transplanted into NSG mice using the IBMI technique, as previously reported[1-4]. At Week 20, the mice were euthanized, and the human CD45$^+$ cell repopulation ability in mouse BM was analyzed by FCM. The mice were scored as positive if more than 0.01 % of the total murine BM cells were human CD45$^+$ cells, as previously reported[1]. The actual repopulation rates of human CD45$^+$ cells in the LDA ranged from 0.06 % to 69.8 %. The frequency of SRC in each of the fractions was calculated using an ELDA web tool[5].

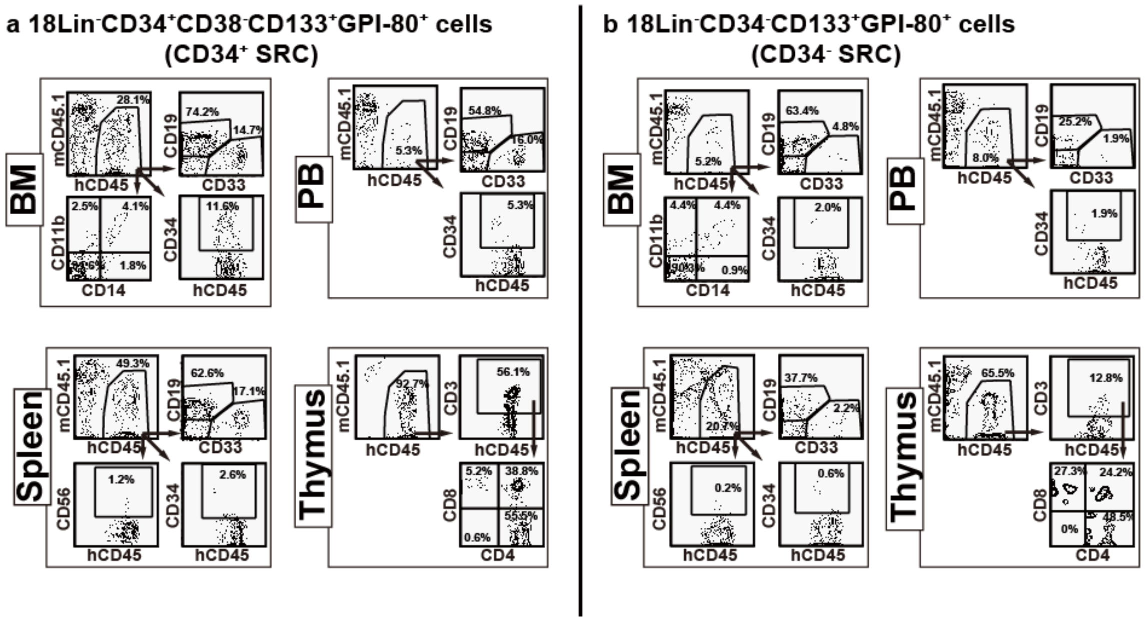

Supplementary Figure 6. Multi-lineage differentiation potential of 18Lin$^-$CD34$^+$CD38$^-$CD133$^+$GPI-80$^+$ cells (CD34$^+$ SRCs [HSCs]) and 18Lin$^-$CD34$^-$CD133$^+$GPI-80$^+$ cells (CD34$^-$ SRCs [HSCs]) in the secondary recipient mice.

Human multi-lineage hematopoietic repopulations in the representative secondary recipient NOG mice engrafted with BMs from the primary recipient mice that received 200 18Lin$^-$CD34$^+$CD38$^-$CD133$^+$GPI-80$^+$ or 200 18Lin$^-$CD34$^-$CD133$^+$GPI-80$^+$ cells, were analyzed. The data clearly demonstrated the secondary multi-lineage repopulation of (**a**) CD34$^+$ SRCs and (**b**) CD34$^-$ SRCs. The expression of CD19, CD33, CD34 (BM, PB, spleen), CD11b and CD14 (BM), CD56 (spleen), and CD3, CD4 and CD8 (thymus) in living human CD45$^+$ cells was analyzed by 6-color FCM. Detailed FCM data, including numbers of experiments, and the mean ± S.D. and ranges of proportions of CD45$^+$ cells and various lineage-positive cells in the BM, PB, spleen and thymus, are presented in **Supplementary Data 2**.

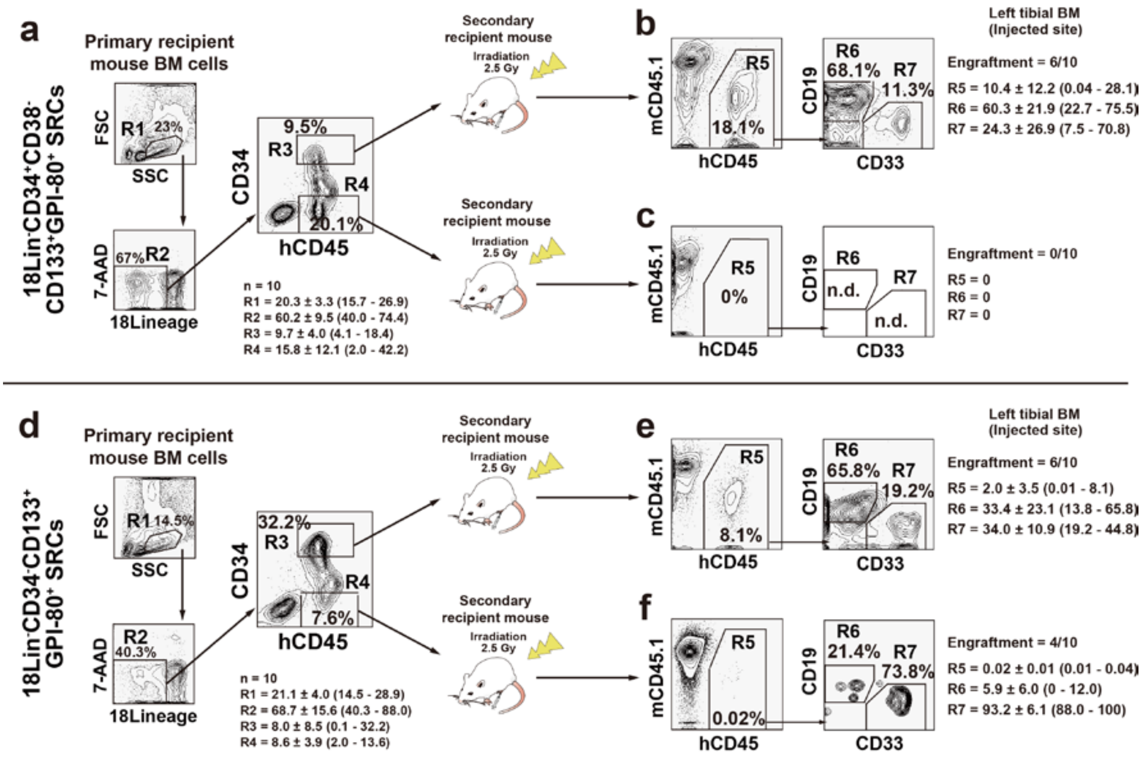

Supplementary Figure 7. Multi-lineage differentiation ability of CD34^{+} and CD34^{-} SRCs generated in primary recipient NOG mice that received CB-derived 18Lin^{-}CD34^{+}CD38^{-}CD133^{+}GPI-80^{+} cells (CD34^{+} SRCs [HSCs]) and 18Lin^{-}CD34^{-}CD133^{+}GPI-80^{+} cells (CD34^{-} SRCs [HSCs])

We transplanted 200 18Lin^{-}CD34^{+}CD38^{-}CD133^{+}GPI-80^{+} and 18Lin^{-}CD34^{-}CD133^{+}GPI-80^{+} cells into primary recipient NOG mice (n=25 for CD34^{+} SRCs and n=23 for CD34^{-} SRCs) (See **Supplementary Data 2a**). At 19-23 weeks after transplantation, we euthanized these mice, and BM cells were obtained for secondary transplantation. We resorted 18Lin^{-}CD34^{+} and 18Lin^{-}CD34^{-} cells in 10 recipient mice that showed high repopulating capacities, as shown in (**a**) and (**d**). Both 18Lin^{-}CD34^{+} and 18Lin^{-}CD34^{-} cells obtained from primary recipient mice that received CD34^{+} and CD34^{-} SRCs were then transplanted into 10 mice each. Multi-lineage human

hematopoietic cell repopulations in representative secondary recipient mice that received (**b**) (**e**) 18Lin$^-$CD34$^+$ and (**c**) (**f**) 18Lin$^-$CD34$^-$ cells generated in primary recipient mice are presented. As shown in **Supplementary Data 2a**, for CD34$^+$ SRCs, 6/10 secondary recipient NOG mice that received 18Lin$^-$CD34$^+$ cells were repopulated with human CD45$^+$ cells with multi-lineage differentiation (**b**). In contrast, none of the mice that received 18Lin$^-$CD34$^-$ cells were repopulated with human CD45$^+$ cells (**c**). For CD34$^-$ SRCs, 6/10 (**e**) and 4/10 (**f**) secondary recipient NOG mice that received 18Lin$^-$CD34$^+$ and 18Lin$^-$CD34$^-$ cells were repopulated with human CD45$^+$ cells with multi-lineage differentiation. Detailed FCM data including numbers of experiments, mean ± S.D. and ranges of proportions of CD45$^+$ cells and various lineage-positive cells in the BM, are presented in the figure and **Supplementary Data 2d**.

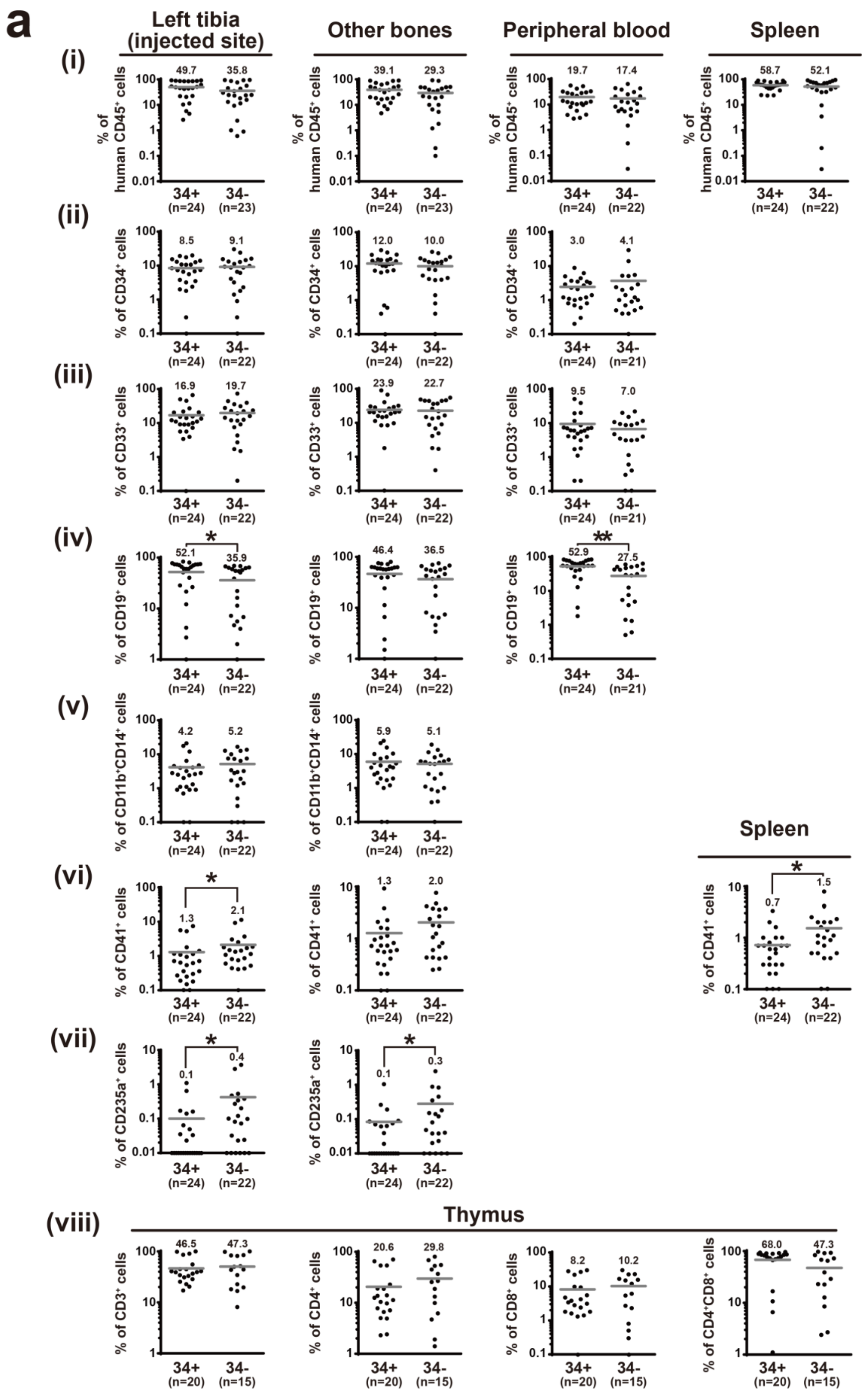
a
Left tibia (injected site)
Other bones
Peripheral blood
Spleen
Thymus
(i)
(ii)
(iii)
(iv)
(v)
(vi)
(vii)
(viii)
% of human CD45+ cells
% of CD34+ cells
% of CD33+ cells
% of CD19+ cells
% of CD11b+CD14+ cells
% of CD41+ cells
% of CD235a+ cells
% of CD3+ cells
% of CD4+ cells
% of CD8+ cells
% of CD4+CD8+ cells
34+
34-
(n=24)
(n=23)
(n=22)
(n=21)
(n=20)
(n=15)
49.7
35.8
39.1
29.3
19.7
17.4
58.7
52.1
8.5
9.1
12.0
10.0
3.0
4.1
16.9
19.7
23.9
22.7
9.5
7.0
52.1
35.9
46.4
36.5
52.9
27.5
4.2
5.2
5.9
5.1
1.3
2.1
1.3
2.0
0.7
1.5
0.1
0.4
0.1
0.3
46.5
47.3
20.6
29.8
8.2
10.2
68.0
47.3
*
**

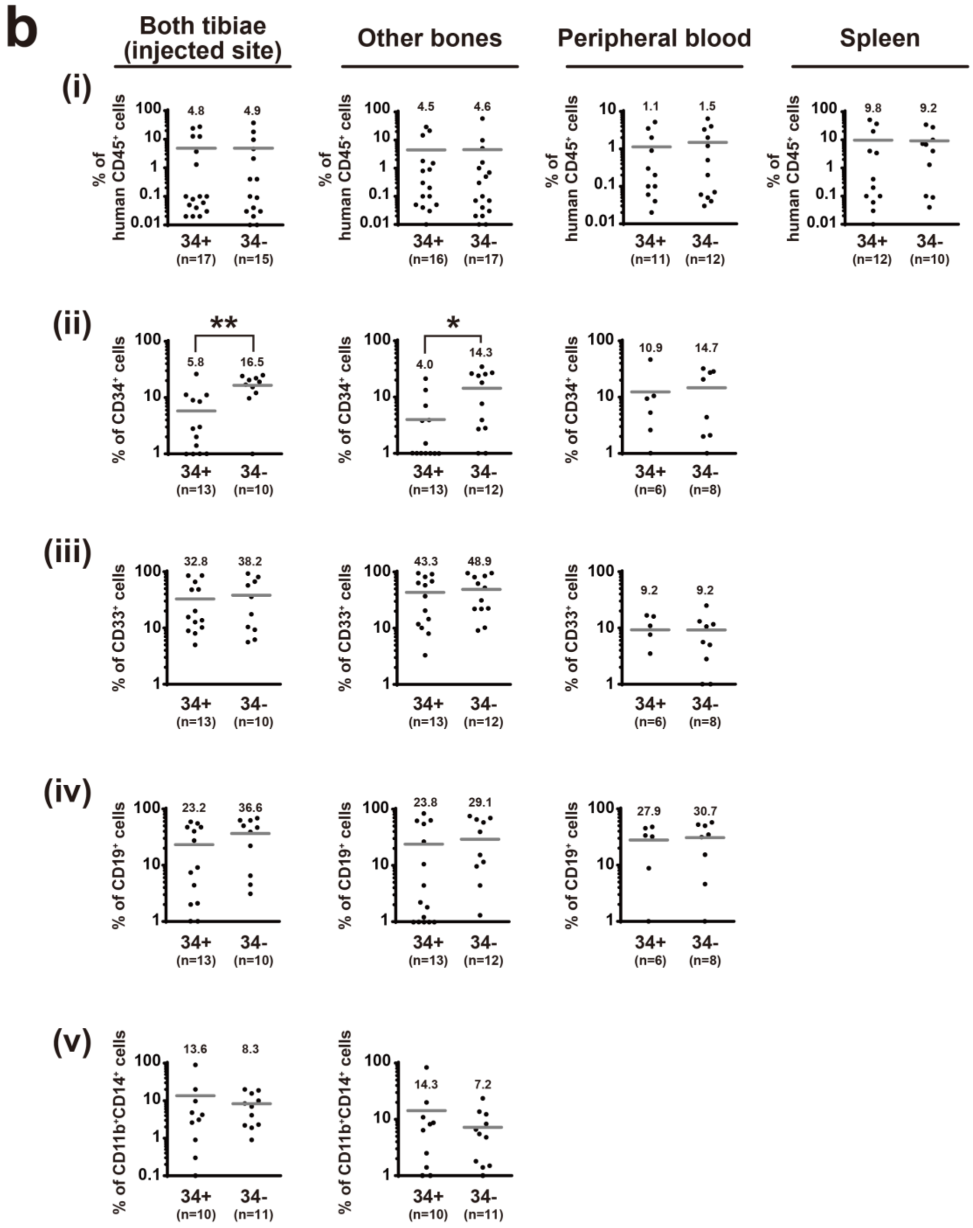

Supplementary Figure 8. *In vivo* multi-lineage differentiation analyses of CD34$^+$ and CD34$^-$ SRCs in the primary and secondary recipient mice.

Human multi-lineage hematopoietic cell repopulation rates in the primary (**a**) and secondary (**b**) recipient mice that received CB-derived 18Lin$^-$CD34$^+$CD38$^-$CD133$^+$GPI-80$^+$ (CD34$^+$ SRCs) and 18Lin$^-$CD34$^-$CD133$^+$GPI-80$^+$ cells (CD34$^-$ SRCs) were quantitatively analyzed. (**a-i**) Human CD45$^+$ cell repopulations in left tibia, other bone,

peripheral blood and spleen of primary recipient mice were analyzed at 20 to 22 weeks after transplantation. (**a-ii** to **a-vii**) The percentages of $CD34^+$, $CD33^+$, $CD19^+$, $CD11b^+CD14^+$, $CD41^+$, and $CD235a^+$ cells in the human $CD45^+$ cells in the primary recipient mouse tissues are shown. (**a-viii**) The percentages of human T-cells in the thymi of the primary recipient mice are shown. (**b-i**) Human $CD45^+$ cell repopulations in both tibiae, other bone, peripheral blood and spleen of secondary recipient mice were analyzed at 19 to 23 weeks after transplantation. (**b-ii** to **b-v**) The percentages of $CD34^+$, $CD33^+$, $CD19^+$ and $CD11b^+CD14^+$ cells in the human $CD45^+$ cells in the secondary recipient mouse tissues are presented. Each dot shows the data from an individual mouse. The horizontal bar represents the mean percentage of marker-positive cell. The numbers of mice analyzed in the specified lineages are depicted in the bottom of each figure. The mean percentage of each data set is also depicted at the top of graph. Statistically significant pairs are denoted by asterisks (*$p < 0.05$, **$p < 0.01$, Mann-Whitney U test).

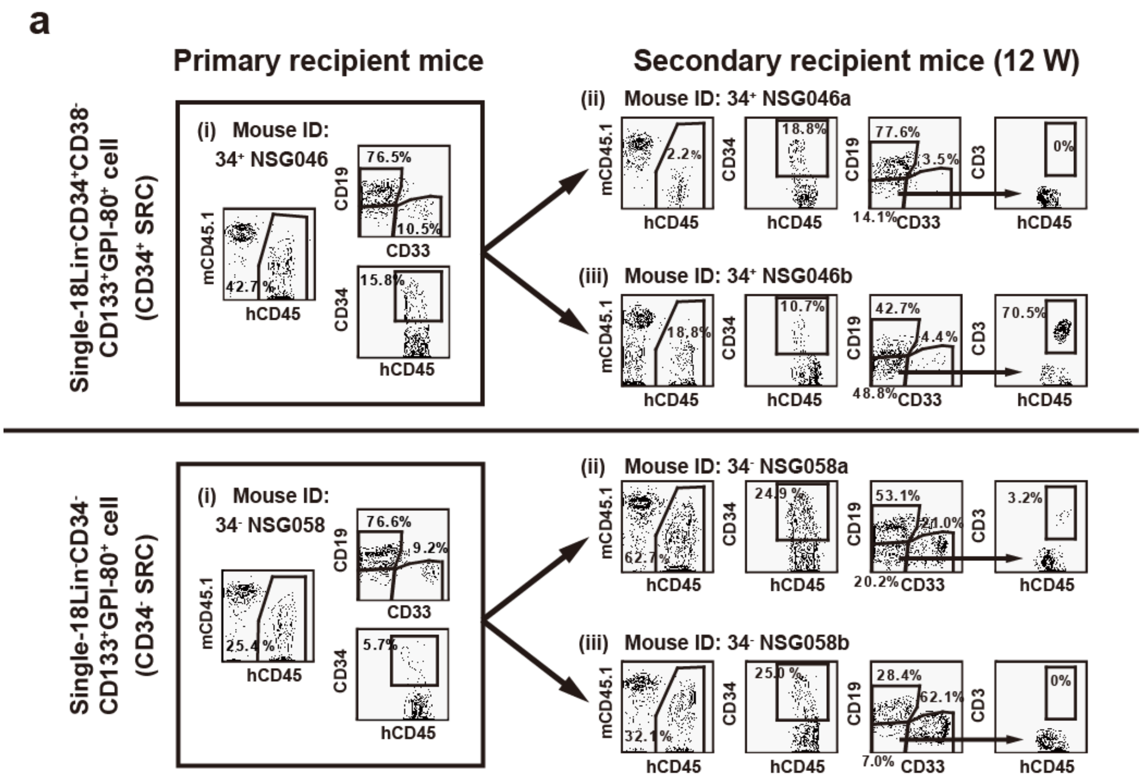
a
Primary recipient mice
Secondary recipient mice (12 W)
Single-18Lin-CD34+CD38-CD133+GPI-80+ cell (CD34+ SRC)
(i) Mouse ID: 34+ NSG046
(ii) Mouse ID: 34+ NSG046a
(iii) Mouse ID: 34+ NSG046b
Single-18Lin-CD34-CD133+GPI-80+ cell (CD34- SRC)
(i) Mouse ID: 34- NSG058
(ii) Mouse ID: 34- NSG058a
(iii) Mouse ID: 34- NSG058b
mCD45.1
hCD45
CD19
CD33
CD34
CD3

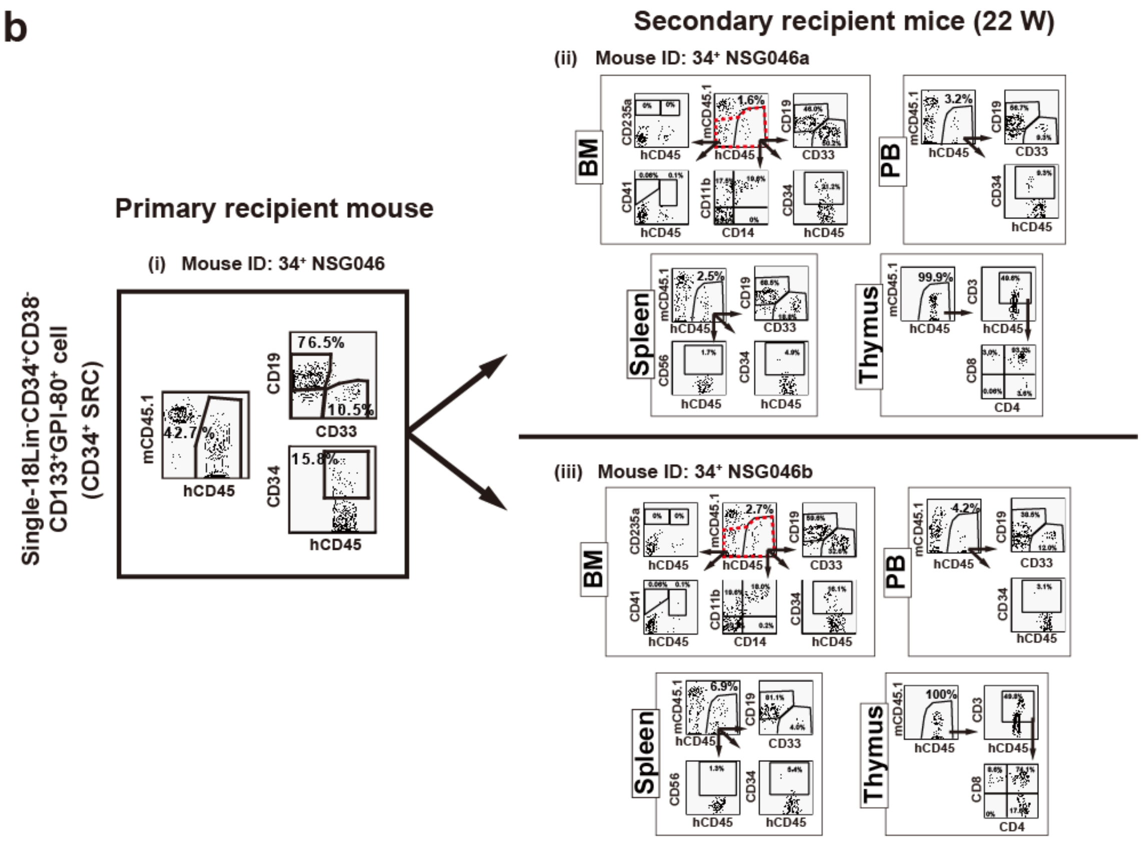
b
Primary recipient mouse
Secondary recipient mice (22 W)
Single-18Lin-CD34+CD38-CD133+GPI-80+ cell (CD34+ SRC)
(i) Mouse ID: 34+ NSG046
(ii) Mouse ID: 34+ NSG046a
(iii) Mouse ID: 34+ NSG046b
BM
PB
Spleen
Thymus

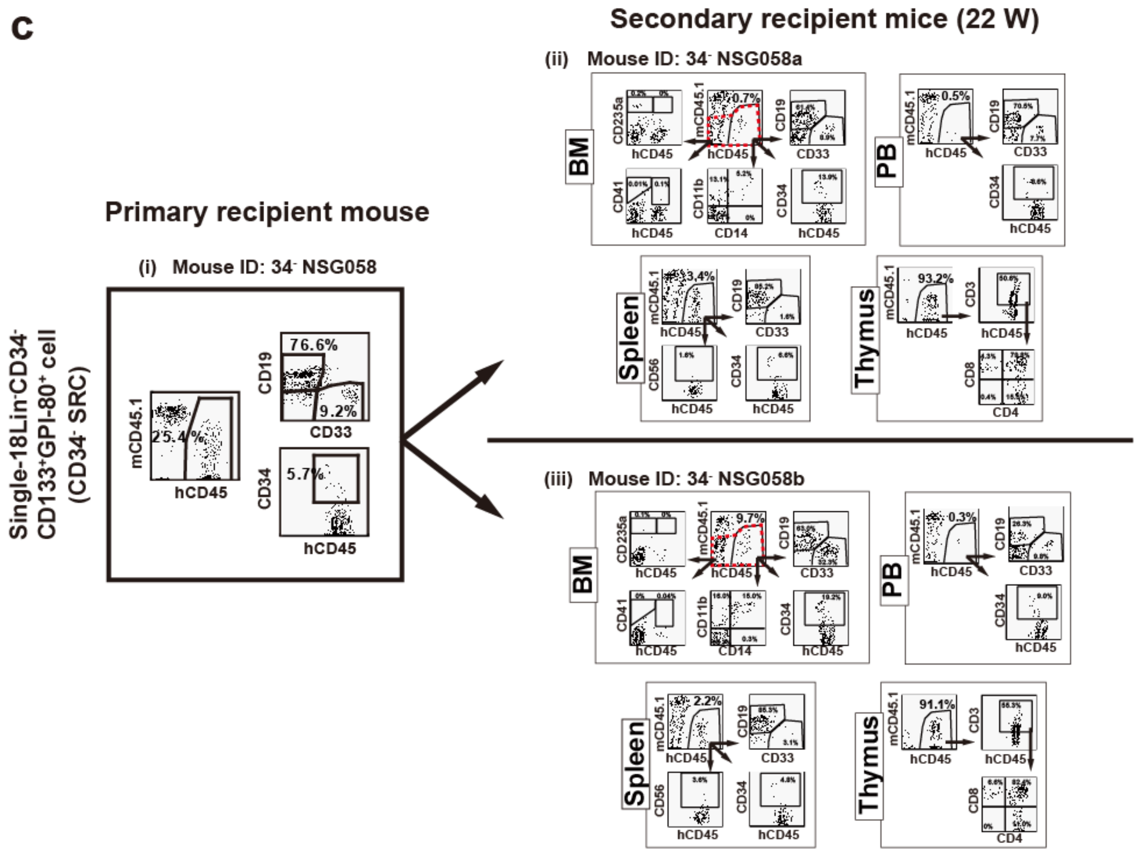
c
Secondary recipient mice (22 W)
(ii) Mouse ID: 34- NSG058a
BM
PB
Spleen
Thymus
Primary recipient mouse
(i) Mouse ID: 34- NSG058
Single-18Lin-CD34-
CD133+GPI-80+ cell
(CD34- SRC)
mCD45.1
hCD45
25.4%
CD19
CD33
76.6%
9.2%
CD34
5.7%
(iii) Mouse ID: 34- NSG058b
CD235a
CD41
CD11b
CD14
CD56
CD3
CD8
CD4
0.7%
0.5%
3.4%
93.2%
9.7%
0.3%
2.2%
91.1%

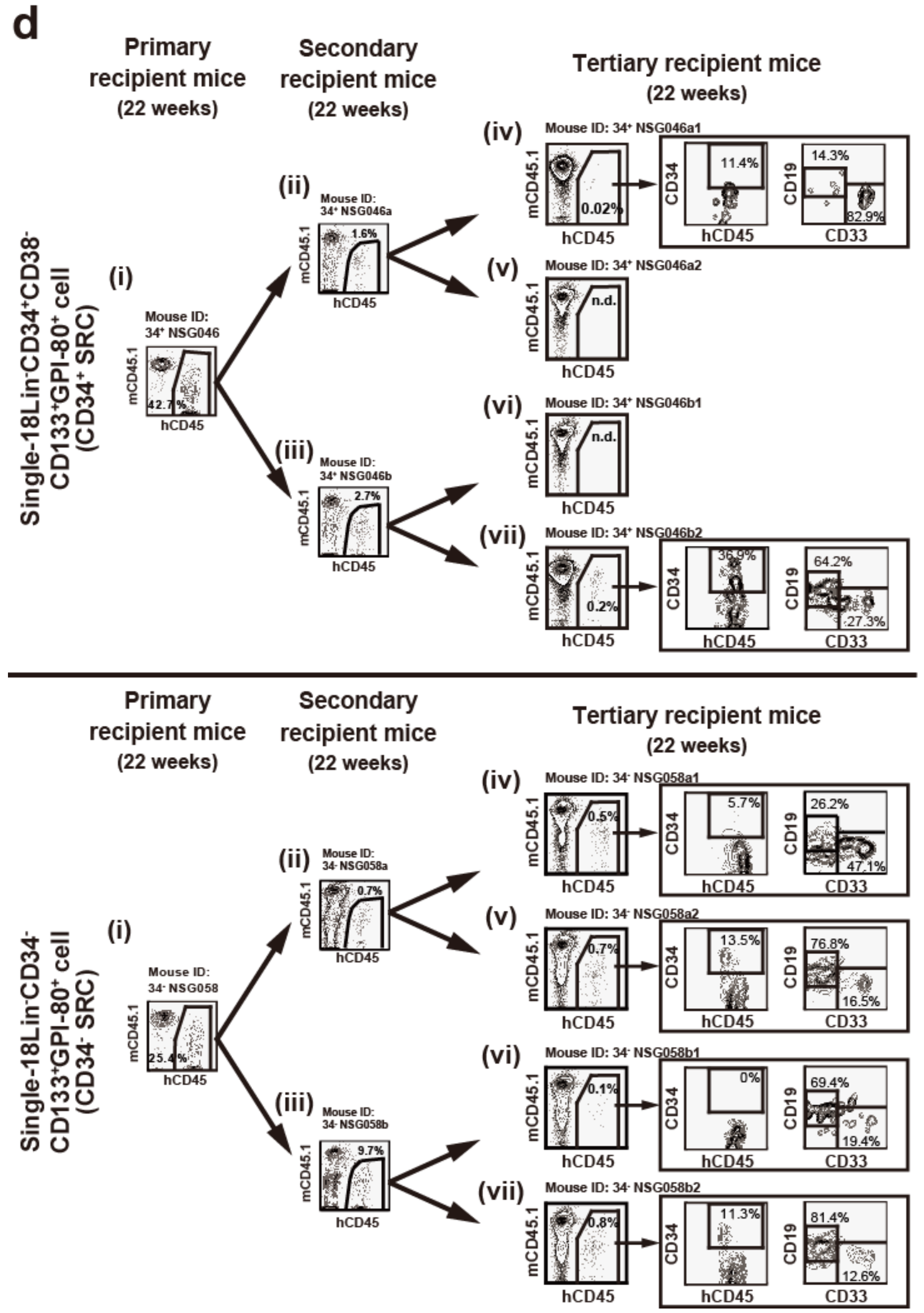

Supplementary Figure 9. Evidence for the *in vivo* self-renewing capacities of CD34^{+} and CD34^{-} SRCs (HSCs) taken by single-cell-initiated serial transplantation.

(**a, upper column**) BM cells obtained from primary recipient mouse (ID: 34^{+} NSG046, also shown in **Supplementary Data 3a**) that received a single 18Lin^{-}CD34^{+}CD38^{-}CD133^{+}GPI-80^{+} cell were collected from both of the tibiae and femurs 22 weeks after

transplantation (**i**). A 2/5 volume of BM cells was transplanted into each of 2 secondary recipient NSG mice (**ii** and **iii**). At 12 weeks after transplantation, BM cells were obtained from the left tibia by BM aspiration. The single $CD34^+$ SRC-derived multi-lineage human hematopoietic repopulation was observed in BMs of both of the secondary recipient mice (Mouse ID: 34^+ NSG046a and 34^+ NSG046b). (**a, lower column**) BM cells obtained from the primary recipient mouse (ID: 34^- NSG058, also shown in **Supplementary Data 3a**) that received a single $18Lin^-CD34^-CD133^+GPI\text{-}80^+$ cell were collected from both of the tibiae and femurs (**i**). A 2/5 volume of BM cells was then transplanted into each of 2 secondary recipient NSG mice (**ii** and **iii**). At 12 weeks after transplantation, BM cells were obtained from the left tibia by BM aspiration. The single $CD34^-$ SRC-derived multi-lineage human hematopoietic repopulations were observed in BMs of both of the secondary recipient mice (Mouse ID: 34^- NSG058a and 34^- NSG058b). (**b**, **c**) At 22 weeks after transplantation, all four above-mentioned secondary recipient mice were euthanized, and the human cell repopulation in the BM, PB, spleen and thymus were precisely analyzed. Both secondary recipient mice showed multi-lineage reconstitution similar to the primary recipient mice, including $CD34^+$ stem/progenitor cells, $CD19^+$ B-lymphoid, $CD33^+$ and $CD11b^+$ myeloid, $CD14^+$ monocytic and $CD41^+$ megakaryocytic cells. Interestingly, $CD235a^+$ erythroid cells were detectable only in the secondary recipient mice that received BM cells from $CD34^-$ SRCs. The presence of $CD41^+$ and $CD235a^+$ cells was analyzed using a human $CD45^{+/-}$ cell gate (depicted by red dotted lines). We also detected $CD56^+$ NK cells in the spleen and $CD3^+$ and CD4/CD8 single/double-positive cells in the thymus. (**d**) A 2/5 volume of remaining BM cells obtained from the two secondary recipient mice that originally received single $CD34^+$ SRCs (mouse ID: 34^+ NSG046a and 34^+ NSG046b, upper

column) was transplanted into 2 tertiary recipient NSG mice (4 mice in total). Simultaneously, a 2/5 volume of remaining BM cells obtained from the two secondary recipient mice that originally received single $CD34^-$ SRC (mouse ID: 34^- NSG058a and 34^- NSG058b, lower column) was transplanted into 2 tertiary recipient NSG mice (4 mice in total). At 22 weeks after transplantation, all tertiary recipient mice were euthanized and multi-lineage human hematopoietic cell repopulations in the mouse BMs were analyzed. Single $CD34^+$ and $CD34^-$ SRC initiated tertiary multi-lineage human cell repopulations were observed in 2/4 and 4/4 mice, respectively.

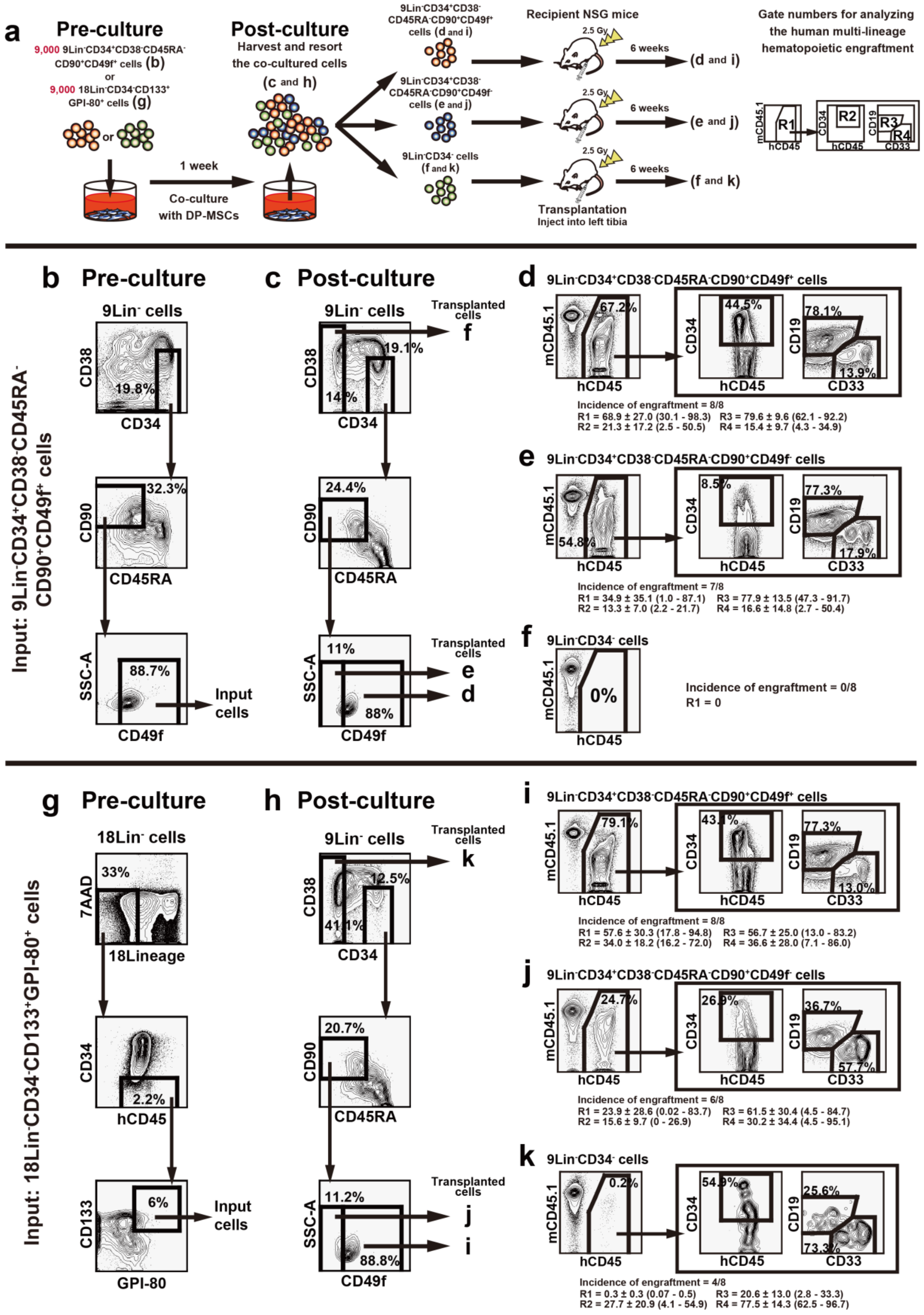

a
Pre-culture
9,000 9Lin-CD34+CD38-CD45RA-CD90+CD49f+ cells (b)
or
9,000 18Lin-CD34-CD133+GPI-80+ cells (g)
1 week
Co-culture with DP-MSCs
Post-culture
Harvest and resort the co-cultured cells
(c and h)
9Lin-CD34+CD38-CD45RA-CD90+CD49f+ cells (d and i)
9Lin-CD34+CD38-CD45RA-CD90+CD49f- cells (e and j)
9Lin-CD34- cells (f and k)
Recipient NSG mice
2.5 Gy
6 weeks
Transplantation
Inject into left tibia
Gate numbers for analyzing the human multi-lineage hematopoietic engraftment
R1 R2 R3 R4
mCD45.1 hCD45 CD34 CD19 CD33
Input: 9Lin-CD34+CD38-CD45RA-CD90+CD49f+ cells
b Pre-culture
9Lin- cells
19.8%
32.3%
88.7%
Input cells
c Post-culture
9Lin- cells
Transplanted cells
19.1%
14%
24.4%
11%
88%
CD38 CD34 CD90 CD45RA SSC-A CD49f
d 9Lin-CD34+CD38-CD45RA-CD90+CD49f+ cells
67.2% 44.5% 78.1% 13.9%
Incidence of engraftment = 8/8
R1 = 68.9 ± 27.0 (30.1 - 98.3) R3 = 79.6 ± 9.6 (62.1 - 92.2)
R2 = 21.3 ± 17.2 (2.5 - 50.5) R4 = 15.4 ± 9.7 (4.3 - 34.9)
e 9Lin-CD34+CD38-CD45RA-CD90+CD49f- cells
54.8% 8.5% 77.3% 17.9%
Incidence of engraftment = 7/8
R1 = 34.9 ± 35.1 (1.0 - 87.1) R3 = 77.9 ± 13.5 (47.3 - 91.7)
R2 = 13.3 ± 7.0 (2.2 - 21.7) R4 = 16.6 ± 14.8 (2.7 - 50.4)
f 9Lin-CD34- cells
0%
Incidence of engraftment = 0/8
R1 = 0
Input: 18Lin-CD34-CD133+GPI-80+ cells
g Pre-culture
18Lin- cells
33%
7AAD 18Lineage
2.2%
CD34 hCD45
6%
CD133 GPI-80
Input cells
h Post-culture
9Lin- cells
12.5%
41.1%
20.7%
11.2%
88.8%
i 9Lin-CD34+CD38-CD45RA-CD90+CD49f+ cells
79.1% 43.1% 77.3% 13.0%
Incidence of engraftment = 8/8
R1 = 57.6 ± 30.3 (17.8 - 94.8) R3 = 56.7 ± 25.0 (13.0 - 83.2)
R2 = 34.0 ± 18.2 (16.2 - 72.0) R4 = 36.6 ± 28.0 (7.1 - 86.0)
j 9Lin-CD34+CD38-CD45RA-CD90+CD49f- cells
24.7% 26.9% 36.7% 57.7%
Incidence of engraftment = 6/8
R1 = 23.9 ± 28.6 (0.02 - 83.7) R3 = 61.5 ± 30.4 (4.5 - 84.7)
R2 = 15.6 ± 9.7 (0 - 26.9) R4 = 30.2 ± 34.4 (4.5 - 95.1)
k 9Lin-CD34- cells
0.2% 54.9% 25.6% 73.3%
Incidence of engraftment = 4/8
R1 = 0.3 ± 0.3 (0.07 - 0.5) R3 = 20.6 ± 13.0 (2.8 - 33.3)
R2 = 27.7 ± 20.9 (4.1 - 54.9) R4 = 77.5 ± 14.3 (62.5 - 96.7)

Supplementary Figure 10. Co-cultures of CB-derived 18Lin$^-$CD34$^-$CD133$^+$GPI-80$^+$ cells (CD34$^-$ HSCs) and 9Lin$^-$CD34$^+$CD38$^-$CD45RA$^-$CD90$^+$CD49f$^+$ cells (CD34$^+$ HSCs) with human BM-derived CD271$^+$SSEA-4$^+$ MSCs (DP MSCs)

(**a**) A schematic illustration of SRC assay for 9Lin$^-$CD34$^+$CD38$^-$CD45RA$^-$CD90$^+$CD49f$^{+/-}$ and 9Lin$^-$CD34$^-$ cells generated from 9Lin$^-$CD34$^+$CD38$^-$CD45RA$^-$CD90$^+$CD49f$^+$ and 18Lin$^-$CD34$^-$CD133$^+$GPI-80$^+$ cells. (**b**) 9Lin$^-$CD34$^+$CD38$^-$CD45RA$^-$CD90$^+$CD49f$^+$ and (**g**) 18Lin$^-$CD34$^-$CD133$^+$GPI-80$^+$ cells were isolated from human CB. Subsequently, 9,000 9Lin$^-$CD34$^+$CD38$^-$CD45RA$^-$CD90$^+$CD49f$^+$ and 9,000 18Lin$^-$CD34$^-$CD133$^+$GPI-80$^+$ cells (3,000 cells/well in triplicate culture) were co-cultured with DP MSCs supplemented with a cocktail of cytokines as described in the **Methods** section. (**c** and **h**) After seven days of co-culture, cultured cells were harvested and 9Lin$^-$CD34$^+$CD38$^-$CD45RA$^-$CD90$^+$CD49f$^{+/-}$ and 9Lin$^-$CD34$^-$ cells maintained/generated from 9Lin$^-$CD34$^+$CD38$^-$CD45RA$^-$CD90$^+$CD49f$^+$ and 18Lin$^-$CD34$^-$CD133$^+$GPI-80$^+$ cells were sorted and collected by FACS. The collected cells were split into eight equal aliquots and then transplanted into the left tibiae of NSG mice by IBMI. At six weeks after transplantation, the human multi-lineage hematopoietic engraftment in the mice receiving (**d** and **i**) 9Lin$^-$CD34$^+$CD38$^-$CD45RA$^-$CD90$^+$CD49f$^+$, (**e** and **j**) 9Lin$^-$CD34$^+$CD38$^-$CD45RA$^-$CD90$^+$CD49f$^-$ and (**f** and **k**) 9Lin$^-$CD34$^-$ cells was analyzed. The incidence of engraftment and the percentages of gated cells (mean ± S.D. and range) are presented beside each FACS plot. See also **Supplementary Data 4**.

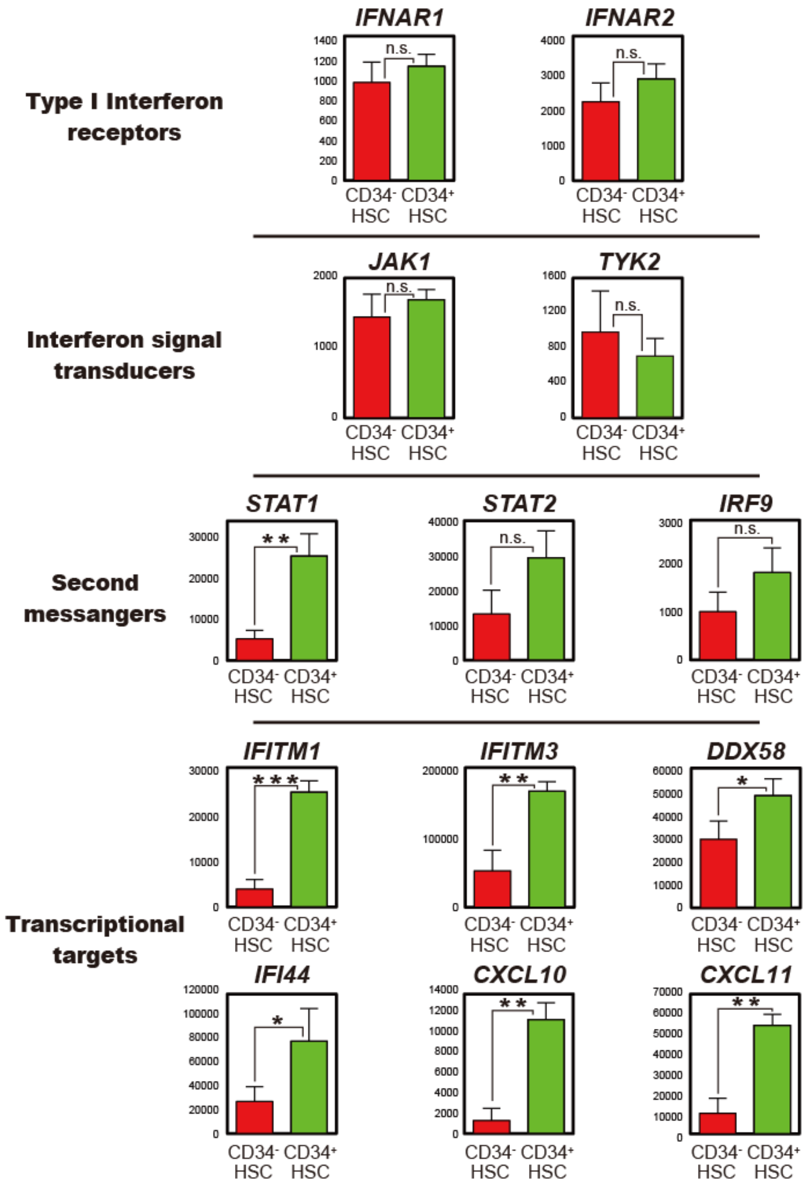

Supplementary Figure 11. A comparison of interferon-related gene expression profiles between CD34$^+$ and CD34$^-$ SRCs (HSCs)

The interferon-related gene expression profiles of CD34$^-$ (red bar) and CD34$^+$ HSC (green bar) were analyzed by microarray. The vertical axis of each graph represents the normalized signal intensity of the target gene obtained from the microarray analysis. The data are presented as the mean values ± S.D. from three independent experiments (*p < 0.05, **p < 0.01, ***p < 0.001, n.s. not significant, two-tailed Student's *t*-test).

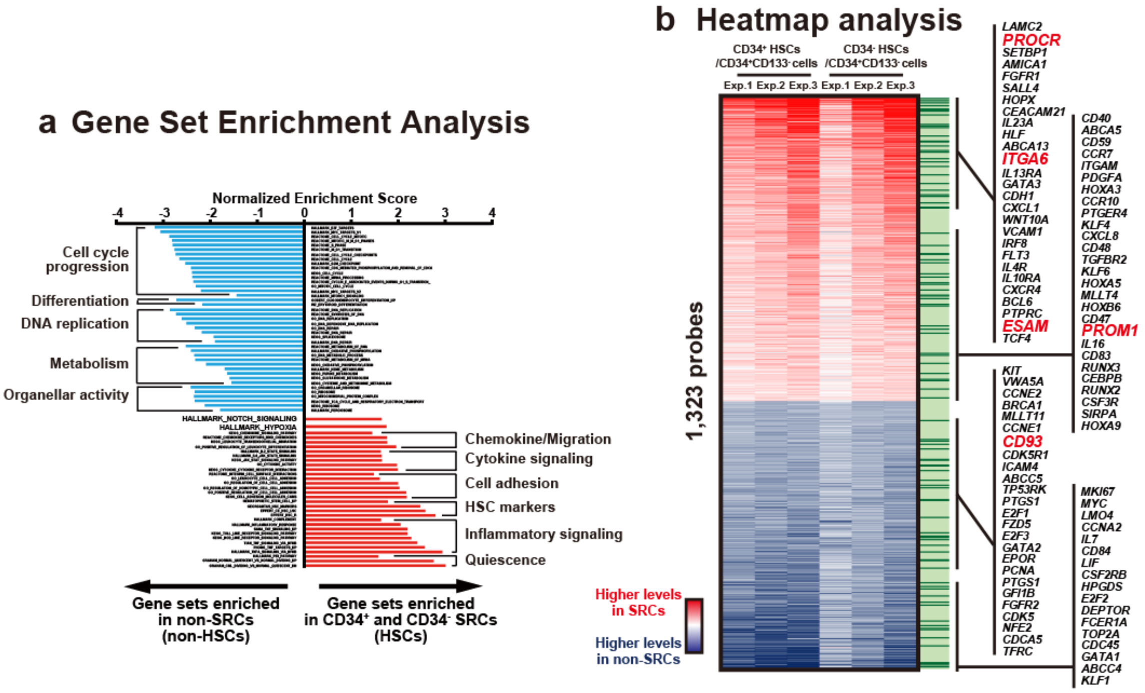

Supplementary Figure 12. A comparison of the gene expression profiles between CD34$^+$ and CD34$^-$SRCs (HSCs) and 18Lin$^-$CD34$^+$CD133$^-$ cells (non-SRCs) (non-HSCs).

(**a**) The gene expression profiles of CD34$^+$ and CD34$^-$ SRCs (HSCs) and non-SRCs (non-HSCs) were compared by a GSEA using the data from the microarray analysis. The gene sets enriched in the non-SRCs (blue bar) and those enriched in CD34$^+$ and CD34$^-$ SRCs (red bar) with a Normalized Enrichment Score < -1 or > 1 were selected and presented. (**b**) The 1,323 probes that were differently expressed in CD34$^+$ and CD34$^-$ SRCs and non-SRCs were also presented by a heatmap. The hematopoietic cell-related genes were extracted and are shown on the right side. The genes of interest are depicted in red. Detailed lists of the gene in each gene set depicted in the figure can be found in the GSEA Molecular Signatures Database (http://software.broadinstitute.org/gsea/msigdb/index. jsp).

a

Number of input cells* (Input CD34⁻ SRCs)	Type of cells transplanted	Number of sorted cells	Number of cells transplanted /mouse	Incidence of engraftment	% of hCD45⁺ cells in the recipient mouse BM	SRC frequency	95% confidence interval	Estimated numbers of output CD34⁺ SRCs**	Expansion efficiency** (Output/Input)
DMSO (Control) 2,850 × 3 (1,056)	12Lin⁻CD45RA⁻ CD34⁺	273,376	1,000	3/8	0.9 - 23.1	1/2,722	1/1,333 - 1/5,560	921	0.87
			4,000	5/7	0.09 - 84.6				
			16,000	6/6	27.8 - 94.1				
	12Lin⁻CD45RA⁻ CD34⁻	556,500	79,500	0/7	N/A	N/A	N/A	N/A	N/A
SR-1 2,850 × 3 (1,056)	12Lin⁻CD45RA⁻ CD34⁺	543,444	1,000	6/8	2.0 - 79.0	1/ 2,792	1/1,367 - 1/5,703	1,404	1.33
			4,000	3/7	0.02 - 30.7				
			16,000	6/6	44.7 - 91.1				
	12Lin⁻CD45RA⁻ CD34⁻	421,400	60,200	0/7	N/A	N/A	N/A	N/A	N/A
UM171 2,850 × 3 (1,056)	12Lin⁻CD45RA⁻ CD34⁺	909,682	2,000	2/8	0.08 - 2.6	1/ 6,364	1/3,117 - 1/12,992	438	0.42
			8,000	5/7	0.01 - 88.8				
			32,000	6/6	9.0 - 95.6				
	12Lin⁻CD45RA⁻ CD34⁻	191,400	31,900	0/6	N/A	N/A	N/A	N/A	N/A

* 2,850 18Lin⁻CD34⁻CD133⁺GPI-80⁺ cells/well were cultured in triplicate.

** CD34⁺ SRCs generated from input CD34⁻ SRCs were evaluated.

N/A: Not applicable

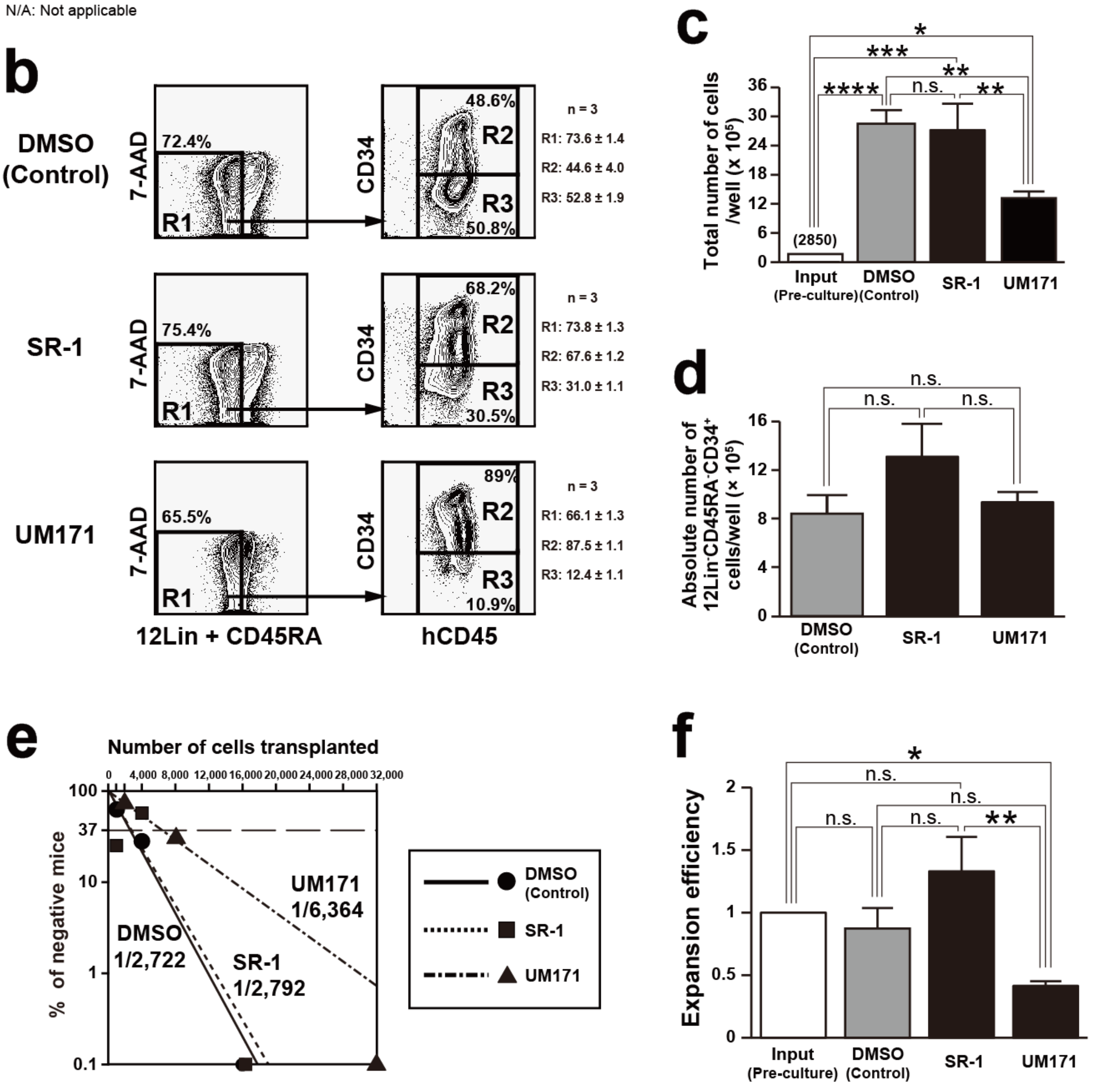

Supplementary Figure 13. ***Ex vivo*** **expansion of CB-derived 18Lin$^-$CD34$^-$CD133$^+$GPI-80$^+$ cells (CD34$^-$ HSCs) using cytokines plus SR-1 or UM171**

(**a**) A summary of the study design and the results of LDA of *in vitro* compound-treated cells. Equal number of 18Lin$^-$CD34$^-$CD133$^+$GPI-80$^+$ cells (2,850 cells/well in triplicate culture) isolated from human CBs were cultured in the presence of SCF, TPO and FL with 0.1% DMSO or 750 nM SR-1 or 35 nM UM171. (**b**) After 7days of *in vitro* culture, the cultured cells were collected and stained with a cocktail of fluorochrome-conjugated monoclonal antibodies. 12Lin$^-$CD45RA$^-$CD34$^+$ cells and 12Lin$^-$CD45RA$^-$CD34$^-$ cells generated from 18Lin$^-$CD34$^-$CD133$^+$GPI-80$^+$ cells in each culture condition were then sorted and collected by FACS. The proportions of cells in each gate are presented on the right side of each plot. The data represents the mean ± S.D.. In order to estimate the frequencies of SRCs in the culture-generated 12Lin$^-$CD45RA$^-$CD34$^+$ cells, various numbers of cells were transplanted into NSG mice, as shown in (**a**). In contrast, all collected 12Lin$^-$CD45RA$^-$CD34$^-$ cells were split into equal aliquots and transplanted into six or seven NSG mice. (**c**) The total numbers of cells produced after culture and (**d**) the absolute numbers of 12Lin$^-$CD45RA$^-$CD34$^+$ cells generated from 18Lin$^-$CD34$^-$CD133$^+$GPI-80$^+$ cells in each well are presented. (**e**) The estimated frequencies of SRCs in the culture-generated 12Lin$^-$CD45RA$^-$CD34$^+$ under each condition are presented. The frequency of SRC in each of the fractions was calculated using an ELDA web tool[5]. Each dot represents the percentages of the negative mice (failed to human cell engraftment) receiving various numbers of 12Lin$^-$CD45RA$^-$CD34$^+$ cells. Filled circle: DMSO (control), filled Square: SR-1, filled triangle: UM171. (**f**) The expansion efficiencies of SRCs under each culture condition were calculated from the numbers of output CD34$^+$ SRCs per those of input CD34$^-$ SRCs. The data are presented as the mean

± S.D. from triplicate culture. (* $p < 0.05$, **$p < 0.01$, *** $p < 0.001$, **** $p < 0.0001$, n.s., not significant, Tukey's multiple comparison procedure).

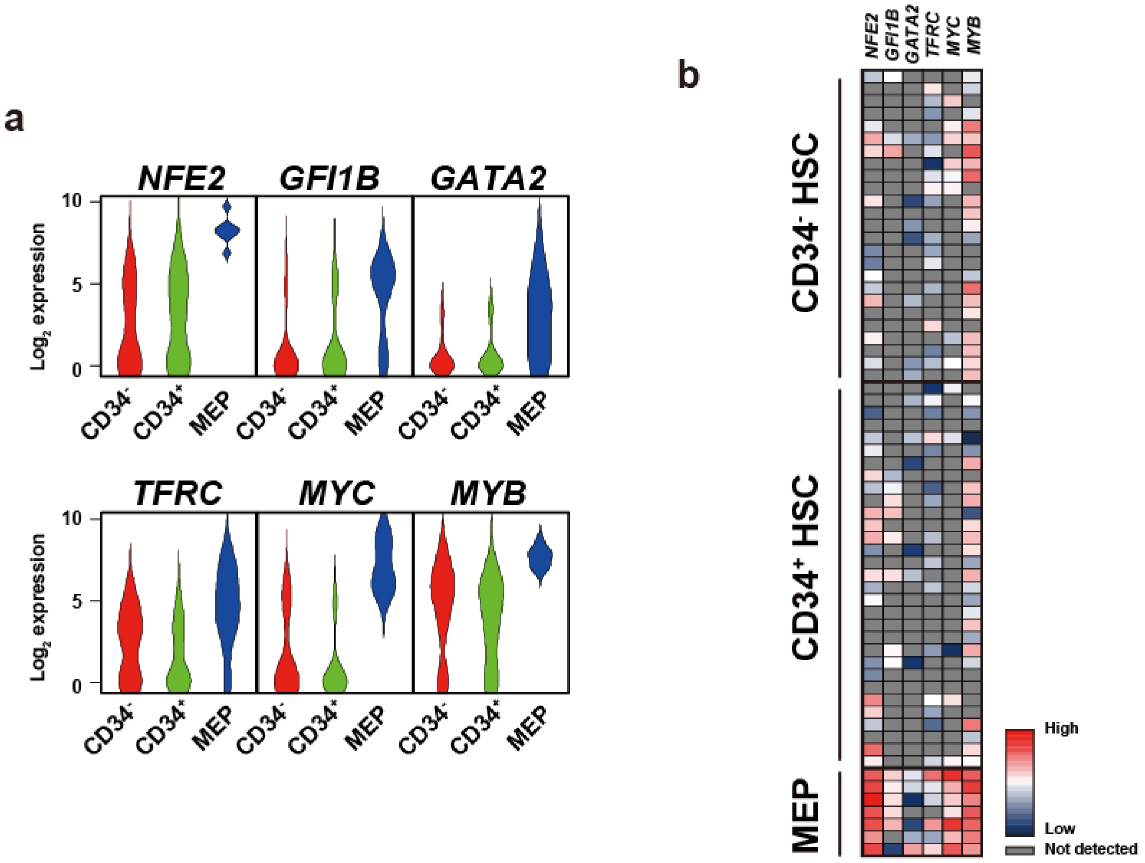

Supplementary Figure 14. A comparison of the gene expression profiles between single CD34$^+$ and CD34$^-$ HSCs and megakaryocyte/erythrocyte progenitors (MEPs).

The gene expression profiles of single CD34$^+$ and CD34$^-$ HSCs and MEPs were compared by single-cell qRT-PCR. (**a**) The genes highly expressed in MEPs were represented by violin plots. (**b**) Expression profiles of the genes shown in (**a**) of single CD34$^+$ and CD34$^-$ HSCs and MEPs is depicted by a heatmap.

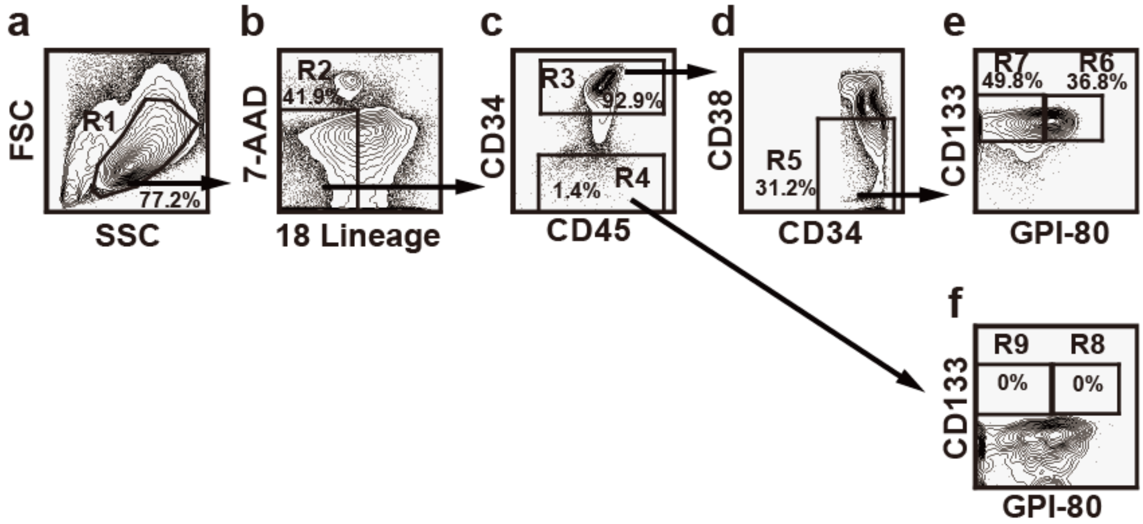

g **Proportions of cells in each gate (%)**
(n = 4, age of volunteer donor: 34-47 years of age [median: 37 years old])

Fraction name	Gate name	Mean ± S.D.	Median	Range
FSC, SSC	R1	60.7 ± 21.8	66.9	31.4 - 77.4
7-AAD$^-$18Lin$^-$	R2	40.2 ± 9.0	38.4	31.8 - 52.0
18Lin$^-$CD45$^+$CD34$^+$	R3	84.4 ± 5.4	82.8	79.9 - 92.2
18Lin$^-$CD45$^+$CD34$^-$	R4	2.7 ± 1.4	2.4	1.4 - 4.6
18Lin$^-$CD45$^+$CD34$^+$CD38$^-$	R5	31.7 ± 17.7	28.5	15.7 - 53.9
18Lin$^-$CD45$^+$CD34$^+$CD38$^-$CD133$^+$GPI-80$^+$	R6	27.0 ± 7.0	29.9	16.7 - 31.5
18Lin$^-$CD45$^+$CD34$^+$CD38$^-$CD133$^+$GPI-80$^-$	R7	60.7 ± 6.9	58.5	55.0 - 70.8
18Lin$^-$CD45$^+$CD34$^-$CD133$^+$GPI-80$^+$	R8	0.1 ± 0.1	0.1	0 - 0.1
18Lin$^-$CD45$^+$CD34$^-$CD133$^+$GPI-80$^-$	R9	0.1 ± 0.1	0	0 - 0.1

Supplementary Figure 15. An analysis of the presence of 18Lin$^-$CD34$^+$CD38$^-$CD133$^+$GPI-80$^+$ and 18Lin$^-$CD34$^-$CD133$^+$GPI-80$^+$ cells in adult BM cells.

The FCM gates for the analysis of BM samples were set in the same way as for the CB samples. (**a**) The forward scatter/side scatter (FSC/SSC) profile of BM-derived Lin$^-$ cells. The R1 gate was set on the blast-lymphocyte window. (**b**) The R2 gate was set on the 18Lin$^-$ living cells. (**c**) The R2 gated cells were subdivided into two fractions: 18Lin$^-$CD45$^+$CD34$^+$ (R3) and CD34$^-$ (R4) cells, according to their expression of CD34. CD34$^+$ and CD34$^-$ cells were defined as follows: the CD34$^+$ fraction contains cells expressing > 5 % of the maximum BV421 fluorescence intensity (FI). The CD34$^-$ level of FI was determined based on the FMO controls. (**d**) The cells residing in the R3 gate were further subdivided into 18Lin$^-$CD45$^+$CD34$^+$CD38$^-$ (R5) cells. The CD38$^-$ fraction

contains cells expressing < 10 % of the maximum PE-Cy7 FI. (**e**) The R5-gated cells were further subdivided into two fractions: 18$Lin^{-}CD45^{+}CD34^{+}CD38^{-}CD133^{+}$GPI-$80^{+}$ (R6) and GPI-80^{-} (R7) cells according to their expression of CD133 and GPI-80. (**f**) The R4-gated cells were further subdivided into two fractions: 18$Lin^{-}CD45^{+}CD34^{-}$ $CD133^{+}$GPI-80^{+} (R8) and GPI-80^{-} (R9) cells. The definitions of $CD133^{+/-}$ and GPI-$80^{+/-}$ cells are the same as for the CB sample. (**g**) Detailed FCM data including numbers of experiments, mean ± S.D., ranges and median of proportions of cells in each gate are presented in the table.

Supplementary References

1. Takahashi, M. *et al.* CD133 is a Positive Marker for a Distinct Class of Primitive Human Cord Blood-derived CD34–negative Hematopoietic Stem Cells. *Leukemia* **28**, 1308-1315 (2014).
2. Wang, J. *et al.* SCID-repopulating cell activity of human cord blood-derived $CD34^-$ cells assured by intra-bone marrow injection. *Blood* **101**, 2924-2931 (2003).
3. Ishii, M. *et al.* Development of a high resolution purification method for precise functional characterization of primitive human cord blood-derived CD34–negative SCID-repopulating cells. *Exp. Hematol.* **39**, 203-213 (2011).
4. Matsuoka, Y. *et al.* GPI-80 expression highly purifies human cord blood-derived primitive CD34-negative hematopoietic stem cells. *Blood* **128**, 2258-2260 (2016).
5. Hu, Y. & Smyth, G.K. ELDA: Extreme limiting dilution analysis for comparing depleted and enriched populations in stem cell and other assays. *J. Immunol. Methods* **347**, 70-78 (2009).
