# Supplementary material for: A revised road map for the commitment of human cord blood CD34-negative hematopoietic stem cells
Source: Nat Commun. 2018 Jun 6;9:2202. doi: 10.1038/s41467-018-04441-z (PMC5989201; doi:10.1038/s41467-018-04441-z)
Supplement: Supplementary file 3 — Description of Additional Supplementary Files [file 41467_2018_4441_MOESM3_ESM.pdf]

Description of Additional Supplementary Files

File Name: Supplementary Data 1
Description: A summary of the limiting dilution analyses.

File Name: Supplementary Data 2
Description: (**a**) A summary of primary and secondary transplantation studies of CB-derived 18Lin^{-}CD34^{+}CD38^{-}CD133^{+}GPI-80^{+} and 18Lin^{-}CD34^{-}CD133^{+}GPI-80^{+} cells. (**b**) A summary of the multi-lineage differentiation potentials of 18Lin^{-}CD34^{+}CD38^{-}CD133^{+}GPI-80^{+} and 18Lin^{-}CD34^{-}CD133^{+}GPI-80^{+} SRCs in the primary recipient mice. (**c**) A summary of the multi-lineage differentiation potentials of 18Lin^{-}CD34^{+}CD38^{-}CD133^{+}GPI-80^{+} and 18Lin^{-}CD34^{-}CD133^{+}GPI-80^{+} SRCs in the secondary recipient mice receiving whole BM cells. (**d**) A summary of multi-lineage differentiation potentials of 18Lin^{-}CD34^{+}CD38^{-}CD133^{+}GPI-80^{+} and 18Lin^{-}CD34^{-}CD133^{+}GPI-80^{+} SRCs in the secondary recipient mice receiving resorted 18Lin^{-}CD34^{+} and 18Lin^{-}CD34^{-} cells.

File Name: Supplementary Data 3
Description: (**a**) A summary of single 18Lin^{-}CD34^{+}CD38^{-}CD133^{+}GPI-80^{+} and 18Lin^{-}CD34^{-}CD133^{+}GPI-80^{+} cell transplantation studies. (**b**) A summary of the multi-lineage differentiation potentials of single 18Lin^{-}CD34^{+}CD38^{-}CD133^{+}GPI-80^{+} and 18Lin^{-}CD34^{-}CD133^{+}GPI-80^{+} SRCs in the primary recipient mice. (**c**) A summary of the multi-lineage differentiation potentials of single 18Lin^{-}CD34^{+}CD38^{-}CD133^{+}GPI-80^{+} and 18Lin^{-}CD34^{-}CD133^{+}GPI-80^{+} SRCs in the secondary recipient mice. (**d**) A summary of the multi-lineage differentiation potentials of single 18Lin^{-}CD34^{+}CD38^{-}CD133^{+}GPI-80^{+} and 18Lin^{-}CD34^{-}CD133^{+}GPI-80^{+} SRCs in the tertiary recipient mice.

File Name: Supplementary Data 4
Description: A summary of the repopulation activities of 9Lin^{-}CD34^{+}CD38^{-}CD45RA^{-}CD90^{+}49f$^{+/-}$ and 9Lin^{-}CD34^{-} cells generated from CD34^{+} and CD34^{-} SRCs after co-culture with DP MSCs.

File Name: Supplementary Data 5
Description: List of antibodies used in this study.

File Name: Supplementary Data 6
Description: The immunophenotypes of target cells for single-cell PCR.

File Name: Supplementary Data 7
Description: Primer sets used for single-cell real-time RT-PCR.
